# Long-term outcomes of dialysis in patients with chronic kidney disease and new-onset atrial fibrillation: A population-based cohort study

**DOI:** 10.1371/journal.pone.0222656

**Published:** 2019-09-19

**Authors:** Tung-Wei Hung, Jing-Yang Huang, Gwo-Ping Jong

**Affiliations:** 1 Division of Nephrology, Department of Internal Medicine, Chung Shan Medical University Hospital and Chung Shan Medical University, Taichung, Taiwan, ROC; 2 Department of Medical Research, Chung Shan Medical University Hospital, Taichung, Taiwan, ROC; 3 Division of Cardiology, Department of Internal Medicine, Chung Shan Medical University Hospital and Chung Shan Medical University, Taichung, Taiwan, ROC; Thomas Jefferson University, UNITED STATES

## Abstract

**Background:**

Chronic kidney disease (CKD) is associated with substantial cardiovascular morbidity. Atrial fibrillation (AF) is a prevalent arrhythmia that increases the risk of both stroke and cardiovascular mortality. Information about the mortality risk among patients with advanced CKD and new-onset AF (NAF) in the presence and absence of dialysis is important. However, the association between advanced CKD and NAF in patients with and without dialysis is unclear.

**Objective:**

To investigate long-term outcomes of the association between advanced CKD and NAF in patients with and without dialysis.

**Methods:**

We conducted a nested case-control study based on the National Health Insurance Program in Taiwan. Each participant aged 20 years and older who had CKD with dialysis from 2000 to 2013 was assigned to the dialysis group, whereas sex-, age-, CKD duration-, and index date-matched participants without dialysis were randomly selected and assigned to the non-dialysis group. We used the Cox regression model to estimate the hazard ratio (HR) with the 95% confidence interval (CI) for mortality in CKD patients with combined dialysis and NAF. Patients with neither NAF nor dialysis served as the reference group.

**Results:**

We identified 3,673 dialysis cases and 7,346 Non-dialysis matched controls for enrolment in the study. The crude mortality rates were 3.3 (95% CI: 3.1–3.5), 10.98 (95% CI: 9.3–13.0), 9.2 (95% CI: 8.7–10.0), and 18.0 (95% CI: 15.4–21.2) in the [Non-dialysis, non-NAF], [Non-dialysis, NAF], [Dialysis, non-NAF], and [Dialysis, NAF] groups, respectively. After adjustment for age, gender, and co-morbidities, the aHRs were 2.0 (95% CI: 1.7–2.3), 2.7 (95% CI: 2.5–2.9), and 3.5 (95% CI: 2.9–4.1) in the [Non-Dialysis, NAF], [Dialysis, non-NAF], and [Dialysis, NAF] groups compared with the [Non-Dialysis, non-NAF] group, respectively. The Kaplan-Meier survival curves showed the highest mortality risk in the [Dialysis, NAF] group among the study groups. Patients with concurrent peritoneal dialysis and AF had the highest mortality risk: aHR = 4.3 (95% CI: 2.3–8.0). However, there was a relatively lower effect of NAF on mortality in patients on dialysis than in patients who were not.

**Conclusions:**

Patients with advanced CKD and NAF had a significantly increased risk of mortality. Dialysis is not risky for patients with concurrent CKD and NAF. Dialysis offers a sufficient survival benefit to be considered as a standard treatment, as indicated by the superior physical status of patients on dialysis.

## Introduction

Atrial fibrillation (AF) is the most common arrhythmia in clinical practice, and its incidence has been increasing over the past few decades [1–4]. The worldwide age-adjusted prevalence of AF, as estimated in the 2010 Global Burden of Disease Study, is 596 per 100,000 men and 373 per 100,000 women, equating to approximately 33 million people [5]. AF is associated with an increased risk of chronic kidney disease (CKD) and developing end-stage renal disease (ESRD) [6], as well as increased mortality [7]. AF is more prevalent in ESRD patients than in age-matched individuals with normal renal function, and patients with concurrent ESRD and AF had higher mortality than those with sinus rhythm [8–10]. The prevalence of AF in patients on hemodialysis (HD) ranges from 3.8% to 27% [11]. The incidence of AF in patients with ESRD is substantially higher than that in the general population [12] and was reported to be higher in patients on HD than in those on peritoneal dialysis (PD) [13,14]. The incidence of AF in these groups varies from 0.5 per 100 person-years to 14.8 per 100 person-years [12]. According to previous studies, the risk factors for AF in ESRD patients were primarily age, followed by hypertension, heart failure, coronary artery disease, peripheral vascular disease, and chronic obstructive pulmonary disease [12,15]. Importantly, AF is associated with a higher mortality risk in dialysis patients [16].

Data on Non-dialysis-dependent and Dialysis-dependent CKD populations are limited. Few reports have addressed the impact of new-onset AF (NAF) in these patients. The impact of NAF on outcomes in CKD patients with and without dialysis has not been determined. In order to promote a better understanding of NAF and CKD, we conducted a nationwide, population-based study to evaluate outcomes among Taiwanese CKD patients to compare the mortality rates for newly developing AF in patients with CKD in the presence and absence of dialysis.

## Materials and methods

### Data sources

This was an observational study using the longitudinal health insurance research dataset (LHIRD) 2000 that had been submitted to the Taiwan Bureau of National Health Insurance from 2000 to 2013. The LHIRD 2000, which includes 1 million beneficiaries who were randomly sampled from the whole population in Taiwan in 2000, is composed of claim records contain inpatient and outpatient visits and examination, prescription, and treatment services. The 2000–2013 dataset of the LHIRD 2000 was collected to complete the study analysis. Therefore, we were able to retrospectively observe the mortality risk among the study groups.

#### Study population

In this study, the first inclusion criterion was patients who visited for CKD (ICD-9-CM codes 582.0, 582.4, 582.8x, 586, 250.4x, 274.1, 403.x1, 404.x2, and 404.x3, corresponding to stage 4 and 5 chronic kidney disease) or ESRD (ICD-9-CM code 585) from 2000 to 2013. In order to improve the accuracy (increase the true-positive rate) of CKD diagnosis, we selected CKD patients who had (1) visited the outpatient clinic two or more times or (2) had been admitted at least once for CKD.

The dialysis cohort was defined as patients who had ESRD (ICD-9-CM code 585) and received dialysis (HD or PD) after being diagnosed with ESRD; the index date was defined as the first date of dialysis. We excluded patients with CKD who were on dialysis when they had cancer (ICD-9: 140–208) or atrial flutter (ICD-9: 427.32) before the index date. The index date of CKD patients not on dialysis was assigned to the corresponding index date of the matched dialysis case.

### Co-morbidities

Atrial fibrillation (AF, ICD-9: 427.31) was the co-existing disease of greatest concern with regard to increasing the mortality risk in patients with CKD. NAF was defined as one outpatient visit or one inpatient admission for NAF after the index date. The other co-morbidities included diabetes mellitus (ICD-9: 250), hypertension (ICD-9: 401–405), hyperlipidemia (ICD-9: 272), coronary artery disease (ICD-9: 410–414), hyperthyroidism (ICD-9: 242), LVH (ICD-9: 429.3), venous thromboembolic disease (ICD-9: 453), autoimmune disease (ICD-9: 714.0, 710.0), cerebral vascular accident (ICD-9: 430–438), chronic obstructive pulmonary disease (ICD-9: 490–492, 494, 496), hemorrhagic stroke (ICD-9: 430–432), and ischemic stroke (ICD-9: 433–436) were also considered in this study.

### Covariates

We hypothesised that several factors may confound the relationship between dialysis and long-term outcomes: age, sex, diabetes mellitus, hypertension, hyperlipidemia, coronary artery disease, hyperthyroidism, LVH, venous thromboembolic disease, autoimmune disease, cerebral vascular accident, chronic obstructive pulmonary disease, hemorrhagic stroke, and ischemic stroke included as a co-variate.

### Ethical approval

This study was reviewed and approved by the Chung Shan Medical University Hospital Review Board. As personal identification information was transformed and encrypted to protect the privacy of study participants, this study was exempted from full review by the Chung Shan Medical University Hospital Review Board (CSUH-18-021).

### Statistical analysis

Time-to-event analysis was performed patients who were on dialysis and those not on dialysis followed up from the index date until death or the end of the study (December 31, 2013). The chi-square test was used to determine the relationships between categorical variables, and the generalized Poisson regression was performed to estimate the mortality rate and its 95% confidence interval (CI). We used the univariate and multivariate Cox regression model to estimate the crude and adjusted hazard ratios (HRs) of mortality for dialysis combined with NAF in patients with CKD. *P* values <0.05 were considered to indicate statistically significant results. All statistical calculations were performed using statistical analysis software, version 9.3 (SAS Institute, Inc., Cary, NC, USA).

## Results

### Baseline characteristics of all patients

**Fig 1:**
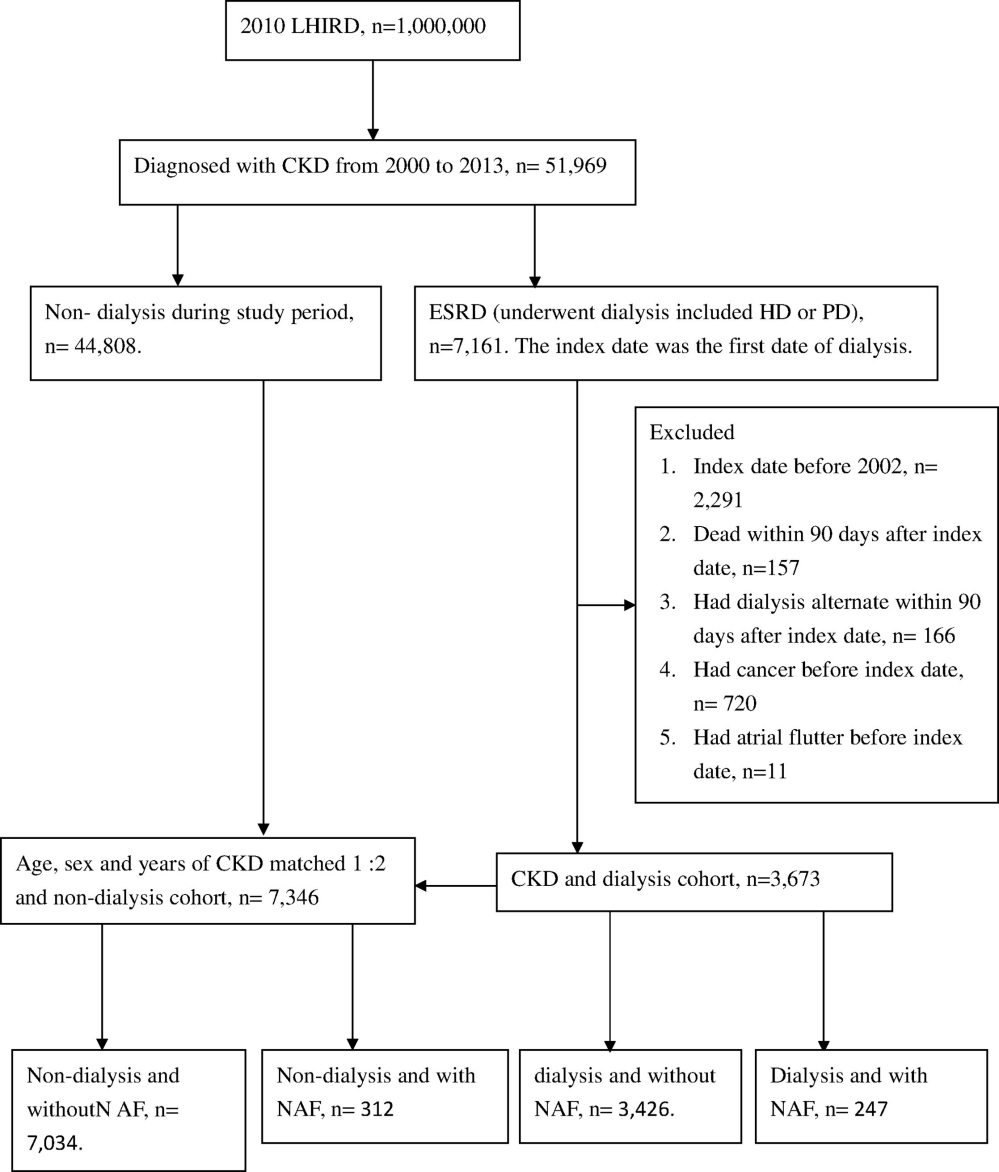
Flowchart of selection of patients for the inclusion in this study.

A total of 51,969 patients identified from LHIRD were diagnosed with CKD from 2000 to 2013. After exclusion and a 1:2 matching procedure, 3,673 dialysis and 7,346 Non-dialysis CKD patients were selected for analysis. There were 247 (6.7%) and 312 (4.2%) AF patients in the dialysis and Non-dialysis groups, respectively (Fig 1).

Table 1 showed the demographic and co-morbidity status in NAF and non-NAF patients stratified by non-ESRD (Non-dialysis) or ESRD (with dialysis). In general, patients with NAF were older with a higher proportion of females, urban dwellers, and co-morbidities. The median follow up times were 53, 33, 39, and 22 months in the [Non-ESRD, non-AF], [Non-ESRD, NAF], [ESRD, non-NAF], and [ESRD, NAF] groups, respectively.

**Table 1:** Baseline characteristic of CKD patients in this study.

	Non-ESRD (non-dialysis)		ESRD (with dialysis)		
	Non-NAF, n = 7,034	With NAF, n = 312	Non-NAF, n = 3,426	With NAF, n = 247	p-value
**Sex**					0.0515
Female	3426(48.71%)	162(51.92%)	1654(48.28%)	140(56.68%)	
Male	3608(51.29%)	150(48.08%)	1772(51.72%)	107(43.32%)	
**Age**					< .0001
<40	288(4.09%)	2(0.64%)	145(4.23%)	0(0.00%)	
40–59	2458(34.94%)	24(7.69%)	1212(35.38%)	29(11.74%)	
60–79	3499(49.74%)	191(61.22%)	1699(49.59%)	146(59.11%)	
> = 80	789(11.22%)	95(30.45%)	370(10.80%)	72(29.15%)	
**Urbanization**					0.0159
Urban	3932(55.9%)	154(49.36%)	1927(56.25%)	125(50.61%)	
Suburban	2217(31.52%)	105(33.65%)	1049(30.62%)	76(30.77%)	
Rural	885(12.58%)	53(16.99%)	450(13.13%)	46(18.62%)	
**Co-morbidity (before index date)**					
Diabetes mellitus	3935(55.94%)	172(55.13%)	2104(61.41%)	156(63.16%)	< .0001
Hypertension	4501(63.99%)	249(79.81%)	3127(91.27%)	229(92.71%)	< .0001
Hyperlipidemia	2865(40.73%)	90(28.85%)	1267(36.98%)	91(36.84%)	< .0001
Coronary artery disease	1515(21.54%)	157(50.32%)	1115(32.55%)	131(53.04%)	< .0001
Hyperthyroidism	76(1.08%)	10(3.21%)	27(0.79%)	6(2.43%)	0.0002
LVH	47(0.67%)	6(1.92%)	39(1.14%)	2(0.81%)	0.0157
Venous thromboembolic disease	30(0.43%)	1(0.32%)	26(0.76%)	2(0.81%)	0.1448
Autoimmune disease	111(1.58%)	4(1.28%)	54(1.58%)	3(1.21%)	0.9465
Cerebral vascular accident	1064(15.13%)	87(27.88%)	688(20.08%)	59(23.89%)	< .0001
COPD	953(13.55%)	77(24.68%)	443(12.93%)	54(21.86%)	< .0001
Hemorrhage stroke	95(1.35%)	5(1.60%)	61(1.78%)	7(2.83%)	0.1246
Ischemic Stroke	744(10.58%)	69(22.12%)	467(13.63%)	47(19.03%)	< .0001

### Relative risk of mortality rates

**Fig 2:**
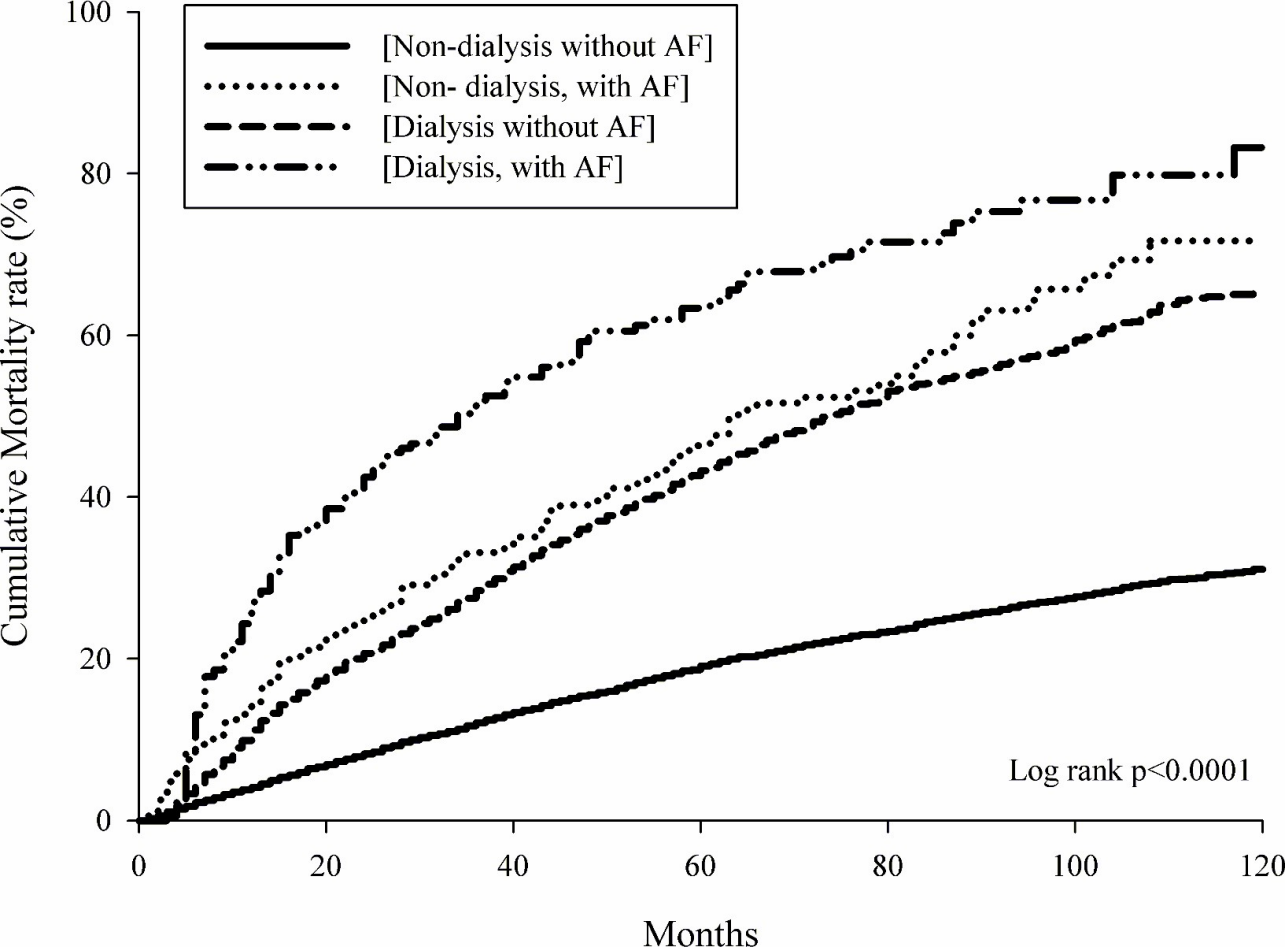
Kaplan-Meier curves for mortality rate in each study group.

Table 2 presents the crude HR for mortality rates, which were 3.3 (95% CI 3.1–3.5), 11.0 (95% CI 9.3–13.0), 9.2 (95% CI 8.7–9.7), and 18.0 (95% CI 15.4–21.2) in the [Non-dialysis, non-NAF], [Non-dialysis, NAF], [Dialysis, non-NAF], and [Dialysis, NAF] groups, respectively. The adjusted HR for mortality rates with co-existing NAF was 1.8 (95% CI. = 1.5–2.2) in the Non-dialysis subset; however, a lower aHR = 1.4 (95% CI 1.1–1.6) was estimated in the Dialysis subset. The *P* value was 0.0005 for the interaction test between AF and dialysis. When compared with the [Non-Dialysis, non-NAF] group, the aHRs were 2.0 (95% CI 1.7–2.3), 2.7 (95% CI 2.5–2.9) and 3.5 (95% CI 2.9–4.1) in the [Non-Dialysis, NAF], [Dialysis, non-NAF], and [Dialysis, NAF] groups, respectively. Finally, Kaplan-Meier survival analysis showed the highest mortality risk among all study groups in the group with dialysis and NAF (Fig 2).

**Table 2:** Relative risk for mortality rate in study groups.

					Adjusted HR*			
Group	N	Pm	Death	Mortality rate#	Stratified	p1	Dummy	p2
**Non- dialysis**								
Non-NAF	7,034	420,668	1,393	3.31(3.14–3.49)	Reference	-	Reference	-
NAF	312	12,930	142	10.98(9.32–12.95)	1.838(1.538–2.197)	< .0001	1.968(1.651–2.346)	< .0001
**Dialysis**								
Non-NAF	3,426	163,076	1,498	9.19(8.73–9.66)	Reference	-		
NAF	247	8,371	151	18.04(15.38–21.16)	1.362(1.147–1.617)	0.0004	2.709(2.508–2.925)	< .0001
					p for interaction	0.0005	3.466(2.920–4.116)	< .0001

Pm, person months

*The stratified aHR was calculated in two separate mode, in non-dialysis and dialysis group, respectively. The dummy variable (reference: non-dialysis and non-NAF) was used in one model.

# per 1,000 Pm

## Discussion

The results of this study showed that CKD patients with NAF had increased mortality, and patients at the time of dialysis initiation with NAF had significantly higher mortality. We also investigated the effect of dialysis for NAF patients compared with Non-dialysis patients in this population and found a lower estimated aHR of mortality equal to 1.4 (95% CI 1.1–1.6) in the Dialysis subset compared with 1.8 (95% CI 1.5–2.2) in the Non-dialysis subset.

The prevalence of AF in the general population ranges from 2.3% to 3.4% [6], and in HD patients it ranges from 3.8% to 27% [11]. CKD is associated with an increased prevalence of NAF. The prevalence of AF was 1.0% among adults without CKD and 2.8%, 2.7%, and 4.2% among adults with stage 1 to 2, stage 3, and stage 4 to 5 CKD, respectively [17]. Dialysis patients had a high incidence of AF [13,18]. In the study, there were 247 (6.7%) and 312 (4.2%) NAF patients with CKD in the Dialysis and Non-dialysis groups, respectively. The current results extend these earlier observations to a large, population-based sample of Taiwanese.

In a previous study, factors associated with AF were older age, extremes (both high and low) of pre-dialysis systolic blood pressure, dialysis modality (hemodialysis vs. PD), and medication use [13]. In the REGARDS study, advanced age, left ventricular hypertrophy, and heart failure were positively associated with the prevalence of AF across all levels of renal function [17]. It was not possible to identify predisposing factors to arrhythmia in the study. We stratified the patients according to gender, age, geographic area, and comorbid conditions. In our study, NAF patients were older with a higher proportion of females, urban dwellers, and co-morbidities, including diabetic mellitus, hypertension, hyperlipidemia, coronary artery disease, hyperthyroidism, left ventricular hypertrophy, cerebral vascular accident, chronic obstructive pulmonary disease, and ischemic stroke.

Previous reports showed that AF was prevalent in patients with CKD who had a poor prognosis, and AF patients on maintenance dialysis were also reported to have a poor prognosis [19–21]. In the previous study, AF was independently associated with higher rates of all-cause mortality [16,22]. CKD is affected by numerous risk factors, and severe co-morbidities such as diabetes, hypertension, and concomitant cardiac diseases such as cardiomyopathies and myocardial infarction must, of course, result in worse outcomes. We investigated the outcome of NAF among advance CKD patients with and without dialysis using the National Health Insurance Research Database. The groups of dialysis patients with AF presented with the highest crude mortality rates 18.0 (95% CI 15.4–21.2), and when compared with the groups of Non-dialysis patients without AF, the adjust HR was 3.466 (95% CI 2.9–4.1). AF was associated with a higher rate of heart failure, which was identified as a significant predictor of mortality [22], while the specific cause of death was not reviewed in our study. In advanced CKD, NAF was associated with a high rate of mortality in the Non-dialysis group. CKD and AF are both associated with a lower quality of life; increased hospitalization rates; and a greater risk of heart failure, stroke, and total mortality. AF and CKD often co-exist; each condition predisposes to the other, and the co-occurrence of these disorders worsens the prognosis relative to either disease alone [20].

The adjusted HR of mortality for co-existing AF was 1.8 (95% CI 1.5–2.2) in the Non-dialysis subset; however, a lower aHR of 1.4 (95% CI 1.1–1.6) was estimated in the dialysis subset. Thus, dialysis is not risky for CKD patients with NAF.

It is also possible that hemodialysis patients present for medical care much more frequently, and asymptomatic AF is more readily detected in them. In advanced CKD patients with significant co-morbidities, clinicians need to consider whether dialysis offers a sufficient survival benefit for it to be a standard treatment, in view of the superior physical status of patients on dialysis. The retrospective nature of this study did not allow performance scores or other indicators of physical status to be collected, although this is an important consideration for future prospective work.

Some limitations of this study need to be emphasized. First, the present study was observational and not randomized; therefore, reported associations should not be construed as causative. Second, we were unable to follow changes in variables over time. Therefore, we could not follow changes in blood pressure, laboratory values, dialysis adequacy, or medications.

## Conclusions

In summary, CKD was associated with an increased prevalence of AF in this large, population-based sample of Taiwanese, and NAF is associated with greater total mortality. The occurrence of AF increased the risk of mortality independently in non-ESRD and ESRD with and without dialysis. Dialysis offers a sufficient survival benefit in patients with CKD and NAF. Thus, dialysis to be considered as a standard treatment, as indicated by the superior physical status of patients on dialysis.
